# 70925 A TL1 team approach to investigate attention and learning at the intracranial network level and assess the effect different cognitive rehabilitation strategies have on measures of attention and learning

**DOI:** 10.1017/cts.2021.572

**Published:** 2021-03-30

**Authors:** Sarah Long, Catherine Tocci, Giridhar Kalamangalam, Ayse Gunduz, William Perlstein

**Affiliations:** 1 University of Florida J. Crayton Pruitt Family Department of Biomedical Engineering, Gainesville, FL; 2 University of Florida Department of Clinical & Health Psychology, Gainesville, FL; 3 University of Florida Department of Neurology, Gainesville, FL

## Abstract

ABSTRACT IMPACT: Access to intracranial recording in our epileptic sample provides a unique opportunity to characterize neurological activation patterns associated with attention and implicit learning; this foundational physiological understanding will serve to better guide cognitive rehabilitation techniques in TBI patients that aim to improve functioning across these cognitive domains. OBJECTIVES/GOALS: 1) Investigate the network level interactions of attention and learning during an attention network task (ANT) and an implicit learning contextual cueing (CC) task. 2) Assess the effect that attention rehabilitation strategies have on behavioral and neural responses pre/post-attentional intervention. METHODS/STUDY POPULATION: This study involves refractory epilepsy patients (rEP) with implanted intracranial electrodes and moderate-to-severe traumatic brain injury (m/sTBI) survivors. In rEP, we are identifying network level modulations of cortical regions via the ANT, which probes components of attention (alerting, orienting, and executive control) and a CC task that probes implicit learning. We hypothesize that modulation of attention and learning can be seen at the neuronal level. In TBI we will assess improvement following two behavioral attention rehabilitation paradigms; and use our results from epileptic patients to guide measurement of treatment-related neuroplastic change via scalp electroencephalography. RESULTS/ANTICIPATED RESULTS: Preliminary behavioral results from the rEP cohort are in line with previous studies and the intracranial data is suggestive of region- and task-specific modulations in memory and attention related systems. Following completion of recruitment, we expect to more concretely identify regions and networks that exhibit modulatory effects associated with attention and implicit learning. Additionally, we anticipate that deficits in attention will be mitigated following training and hypothesize that implicit learning rate will improve in TBI patients as a result of both attentional rehabilitation paradigms. DISCUSSION/SIGNIFICANCE OF FINDINGS: Characterizing intracranial activity in epilepsy patients will give electrophysiology data unattainable in TBI patients. This intracranial perspective will enable us to propose mechanisms of action that may result from our interventions and enable critique of current rehabilitation treatments.

